# Phosphatidylinositide 3-kinase (PI3K) and PI3K-related kinase (PIKK) activity contributes to radioresistance in thyroid carcinomas

**DOI:** 10.18632/oncotarget.11056

**Published:** 2016-08-04

**Authors:** Natalie Burrows, Joseph Williams, Brian A Telfer, Julia Resch, Helen R Valentine, Richard J Fitzmaurice, Amanda Eustace, Joely Irlam, Emily J Rowling, Cuong Hoang-Vu, Catharine M West, Georg Brabant, Kaye J Williams

**Affiliations:** ^1^ Hypoxia and Therapeutics Group, Manchester Pharmacy School, University of Manchester, Manchester, UK; ^2^ Experimental and Clinical Endocrinology, Medizinische Klinik I, Lubeck, Germany; ^3^ Translational Radiobiology Group, University of Manchester, Christie Hospital, NHS Trust, Manchester Academic Health Science Centre, Manchester, UK; ^4^ Department of Histopathology, CSB1, Manchester Royal Infirmary, Manchester, UK; ^5^ Martin Luther University of Halle-Wittenberg, Halle, Salle, Germany; ^6^ Radiotherapy Related Research Group, Manchester Cancer Research Centre, Manchester, UK; ^7^ Current address: School of Clinical Medicine, Cambridge Institute for Medical Research, University of Cambridge, Cambridge Biomedical Campus, Cambridge, UK

**Keywords:** radioresistance, ATM, ATR, DNA-PKcs, PI3K

## Abstract

Anaplastic (ATC) and certain follicular thyroid-carcinomas (FTCs) are radioresistant. The Phosphatidylinositide 3-kinase (PI3K) pathway is commonly hyperactivated in thyroid-carcinomas. PI3K can modify the PI3K-related kinases (PIKKs) in response to radiation: How PIKKs interact with PI3K and contribute to radioresistance in thyroid-carcinomas is unknown. Further uncertainties exist in how these interactions function under the radioresistant hypoxic microenvironment.

Under normoxia/anoxia, ATC (8505c) and FTC (FTC-133) cells were irradiated, with PI3K-inhibition (via GDC-0941 and PTEN-reconstitution into PTEN-null FTC-133s) and effects on PIKK-activation, DNA-damage, clonogenic-survival and cell cycle, assessed. FTC-xenografts were treated with 5 × 2 Gy, ± 50 mg/kg GDC-0941 (twice-daily; orally) for 14 days and PIKK-activation and tumour-growth assessed. PIKK-expression was additionally assessed in 12 human papillary thyroid-carcinomas, 13 FTCs and 12 ATCs.

GDC-0941 inhibited radiation-induced activation of Ataxia-telangiectasia mutated (ATM), ATM-and Rad3-related (ATR) and DNA-dependent protein kinase catalytic subunit (DNA-PKcs). Inhibition of ATM and DNA-PKcs was PI3K-dependent, since activation was reduced in PTEN-reconstituted FTC-133s. Inhibition of PIKK-activation was greater under anoxia: Consequently, whilst DNA-damage was increased and prolonged under both normoxia and anoxia, PI3K-inhibition only reduced clonogenic-survival under anoxia. GDC-0941 abrogated radiation-induced cell cycle arrest, an effect most likely linked to the marked inhibition of ATR-activation. Importantly, GDC-0941 inhibited radiation-induced PIKK-activation in FTC-xenografts leading to a significant increase in time taken for tumours to triple in size: 26.5 ± 5 days (radiation-alone) versus 31.5 ± 5 days (dual-treatment). PIKKs were highly expressed across human thyroid-carcinoma classifications, with ATM scoring consistently lower. Interestingly, some loss of ATM and DNA-PKcs was observed. These data provide new insight into the mechanisms of hypoxia-associated radioresistance in thyroid-carcinoma.

## INTRODUCTION

Anaplastic thyroid carcinomas (ATC) and subsets of follicular thyroid carcinomas (FTC) are aggressive, metastatic and resistant to conventional radiotherapy. Molecular-based targeting of dysregulated signalling cascades including the phosphatidylinositol 3-kinase (PI3K), mitogen-activated protein kinase (MAPK) and related proteins (BRAF, RAS and RET) as single or multi-modal chemotherapeutics look promising for the treatment of differentiated thyroid cancer. However, available clinical results highlight a need for more efficacious combined chemo/radiotherapy treatment modalities for the aggressive, therapy resistant subsets [1–3].

DNA damage induced for example, by radiotherapy activates the DNA damage response (DDR) that involve three major groups of proteins that act in concert to translate the DNA damage signal into a functional response principally involving cell cycle arrest and DNA repair. These proteins sense the DNA lesions and recruit transducer proteins that relay and amplify the damage signal to effector proteins.

A class of proteins rapidly activated in response to DNA damage is the PI3K-related kinases (PIKKs): Members include Ataxia-telangiectasia mutated (ATM), ATM- and Rad3-related (ATR) and DNA-dependent protein kinase catalytic subunit (DNA-PKcs), which function principally as transducer proteins. ATM and DNA-PKcs respond to double-stranded DNA breaks, whilst ATR is activated by single-stranded DNA breaks and stalled replication forks. The PIKKs phosphorylate many proteins that act to maintain genomic integrity by promoting DNA repair, controlling cell cycle progression, transcription and apoptosis (reviewed in [4, 5]). DNA repair may enable tumour cells to survive breaks induced by DNA-damaging treatments [6] such as radiotherapy leading to radioresistance.

The DNA repair capacity of a tumour cell is highly influenced by PIKK activity and their interactions with additional oncogenic signalling cascades. The phosphatidylinositide 3-kinase (PI3K) pathway is commonly hyperactivated in follicular (FTC) and anaplastic thyroid carcinomas (ATC) and contributes to aggressive transition and radioresistance [1, 7]. However, the mechanism of PI3K-mediated radioresistance in thyroid carcinoma is not well understood. In selective tumour types that exhibit hyperactive PI3K signalling, the PIKK proteins are modulated by PI3K in response to DNA damage. The relationship between PI3K and PIKK activation in irradiated cancer cells is complex and differs between tumour types. Modulation of PIKK activity by PI3K is also specific to certain PIKKs. For example, PI3K contributes to ATM activation induced by ionizing radiation in a T cell leukemia cell line, whilst in a study involving lung epithelial carcinoma cells PI3K modulated radiation-induced DNA-PKcs activity and had no effect on ATM [8, 9]. Further complexities exist in that other PI3K-pathway members can be independently activated by PIKKs and vice versa. For example, ATM is required for phosphorylation and full activation of AKT in response to radiation-induced damage, validated in a range of cell lines including fibroblasts derived from ATM knock-out mice and patients with ataxia telangiectasia. Additionally AKT has been reported to act both upstream and downstream of DNA-PKcs to promote tumour cell survival post-radiotherapy [10–13].

Additional uncertainties exist on the relative contribution of these interactions under hypoxia, a tumour condition that contributes significantly to radioresistance. Hypoxic cells are inherently radioresistant as oxygen is required for the generation of DNA damage. The interplay between the PI3K and the Hypoxia-inducible factor (HIF) pathways contributes to tumour progression and metastasis in thyroid carcinoma, and radiotherapy has been shown to enhance metastastic characteristics in models of FTC and ATC via enhanced PI3K/Hypoxia-inducible factor (HIF) signalling [14]. Both pathways contribute significantly to radioresistance of many cancers.

How the apparent activation of PIKKs contributes to radioresistance in thyroid carcinoma has not been evaluated and the relative contribution by the PI3K pathway is unknown. Our main objectives were to a) assess the relationship between PI3K and the PIKKs in irradiated thyroid cancer cells b) determine if PI3K inhibition altered the observed radioresistant phenotypes in cells and xenografts and c) establish a clinical relevance in primary human tumours.

To address this, we used cell lines representative of ATC (8505c) and FTC (FTC-133) that have previously been linked to PI3K activation and aggressive transition in response to radiotherapy in preclinical models [14]. Furthermore, the FTC-133 cell line was used since it originates from an FTC lymph node metastasis and responds poorly to radioiodine [15]. Clinically, subsets of FTC that are phenotypically similar to this cell line may be treated with external beam radiotherapy, similarly to ATC [1, 16]

We first treated cells with GDC-0941 to pharmacologically inhibit PI3K and determined effects on radiation-induced PIKK activation. Further we used a pair of FTC-133 cell lines: one containing a control vector and the other stably transfected with phosphatase and tensin homolog (PTEN), to genetically inhibit the PI3K pathway, since the parental FTC-133 cell line lacks PTEN. In addition to normoxia, studies were performed under anoxia to mimic a condition of high radioresistance and determine any differences in PI3K/PIKK activity between different oxygen tensions. We next assessed if GDC-0941 radiosensitised FTC xenografts and related this to effects on PIKK activation. To ascertain a potential clinical relevance of PIKKs, we assessed total PIKK expression in primary thyroid carcinoma samples representative of PTC, FTC and ATC and in normal thyroid tissue.

## RESULTS

### Radiation-induced PIKK activation is differentially modified by pharmacological (GDC-0941) and genetic inhibition of PI3K in normoxia and anoxia

**Figure 1:**
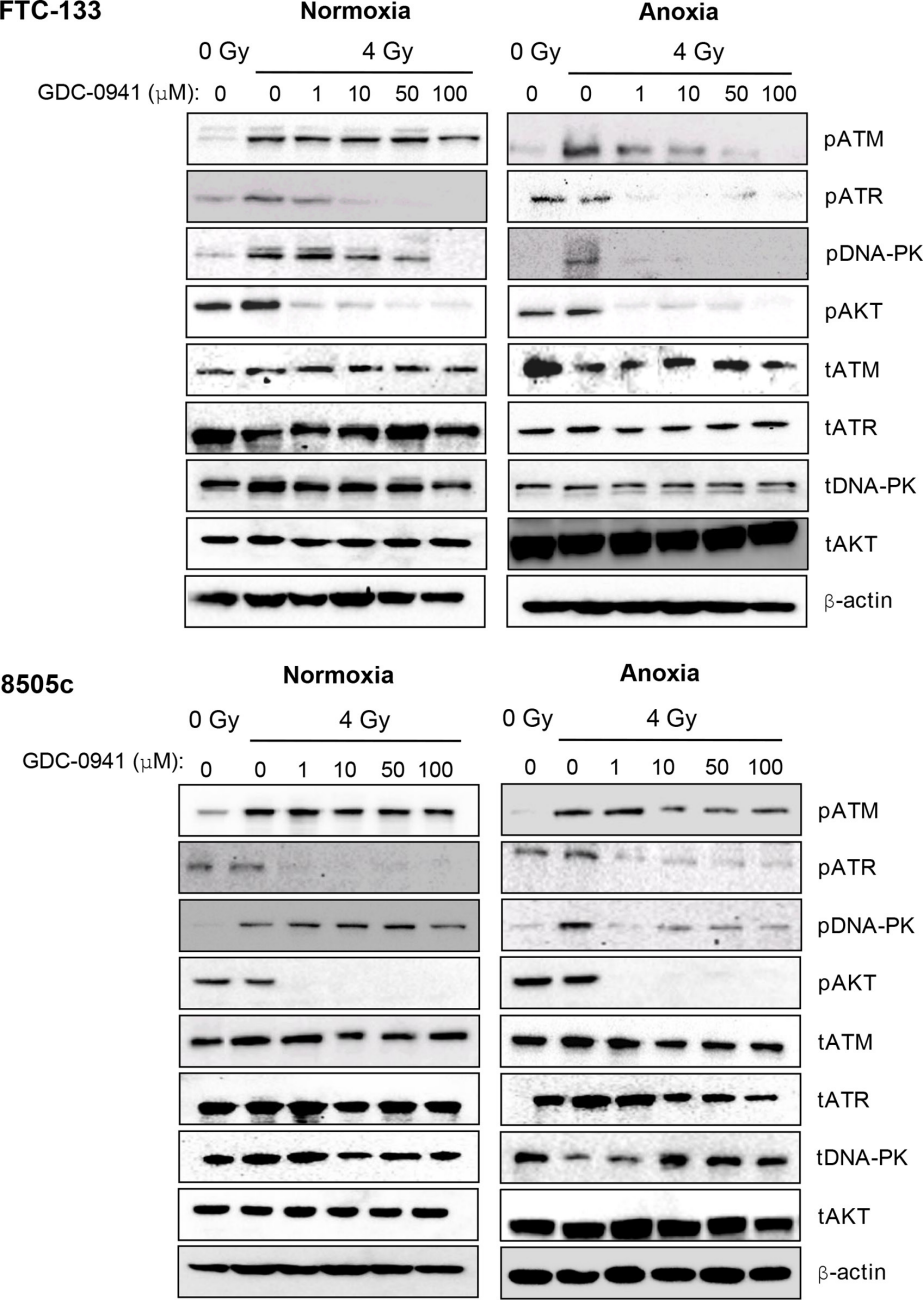
Under anoxia, radiation-induced PIKK activation is markedly inhibited by GDC-0941 PIKK activation was assessed via analysis of phospho(p)PIKK protein expression. In FTC-133 and 8505c cells, GDC-0941 inhibited expression of pATR at concentrations ≤ 1 μM under both normoxia and anoxia. Under normoxia, GDC-0941 inhibited pDNA-PKcs expression at higher concentrations; ≤ 10 μM in FTC-133′s and 100 μM 8505c's. Under anoxia, pDNA-PKcs was much more sensitive to GDC-0941, with almost complete inhibition observed at 1 μM GDC-0941 in both cell lines. Under normoxia, GDC-0941 had little effect on pATM, whilst a clear inhibition was observed in both cell lines under anoxia. Cells were exposed to GDC-0941 in normoxia/anoxia for 18 h, irradiated (4 Gy) whilst remaining in the specified conditions and lysates collected 1 h later for analysis. Cropped blots are representative of 4 independent experiments.

To evaluate whether pharmacological targeting of PI3K influenced PIKK activation we assessed the effect of GDC-0941 on radiation-induced PIKK activation in normoxia and anoxia via analysis of phospho-(p)PIKK expression. We used two thyroid models, the follicular cell line FTC-133 and the anaplastic model 8505c. Under normoxic conditions, pATM was relatively unchanged by increasing doses of GDC-0941 in both FTC-133 and 8505c cells (Figure 1). Under anoxic conditions ATM activation was clearly diminished by GDC-0941 treatment in FTC-133 and 8505c cells. Although the effect in 8505c cells was less marked. pDNA-PKcs was inhibited to a much greater extent by GDC-0941 under anoxia in both cell lines, with 1 μM sufficient to ablate activation. Similar effects in normoxia required doses of > 50 μM or 100 μM in FTC-133 and 8505c cells respectively. In contrast pATR levels after radiation treatment were significantly compromised by 1 μM GDC-0941 in both cell lines under both oxygen conditions (Figure 1). Previous work has demonstrated that GDC-0941 can directly inhibit DNA-PKcs [17]. Consistently pDNA-PKcs was reduced by GDC-0941 alone in FTC-133 cells (data not shown). In unirradiated 8505c cells, pDNA-PKcs was undetectable. pAKT was used as a downstream biomarker for PI3K inhibition. 1 μM GDC-0941 was sufficient to ablate pAKT in both cell lines across conditions (Figure 1).

**Figure 2:**
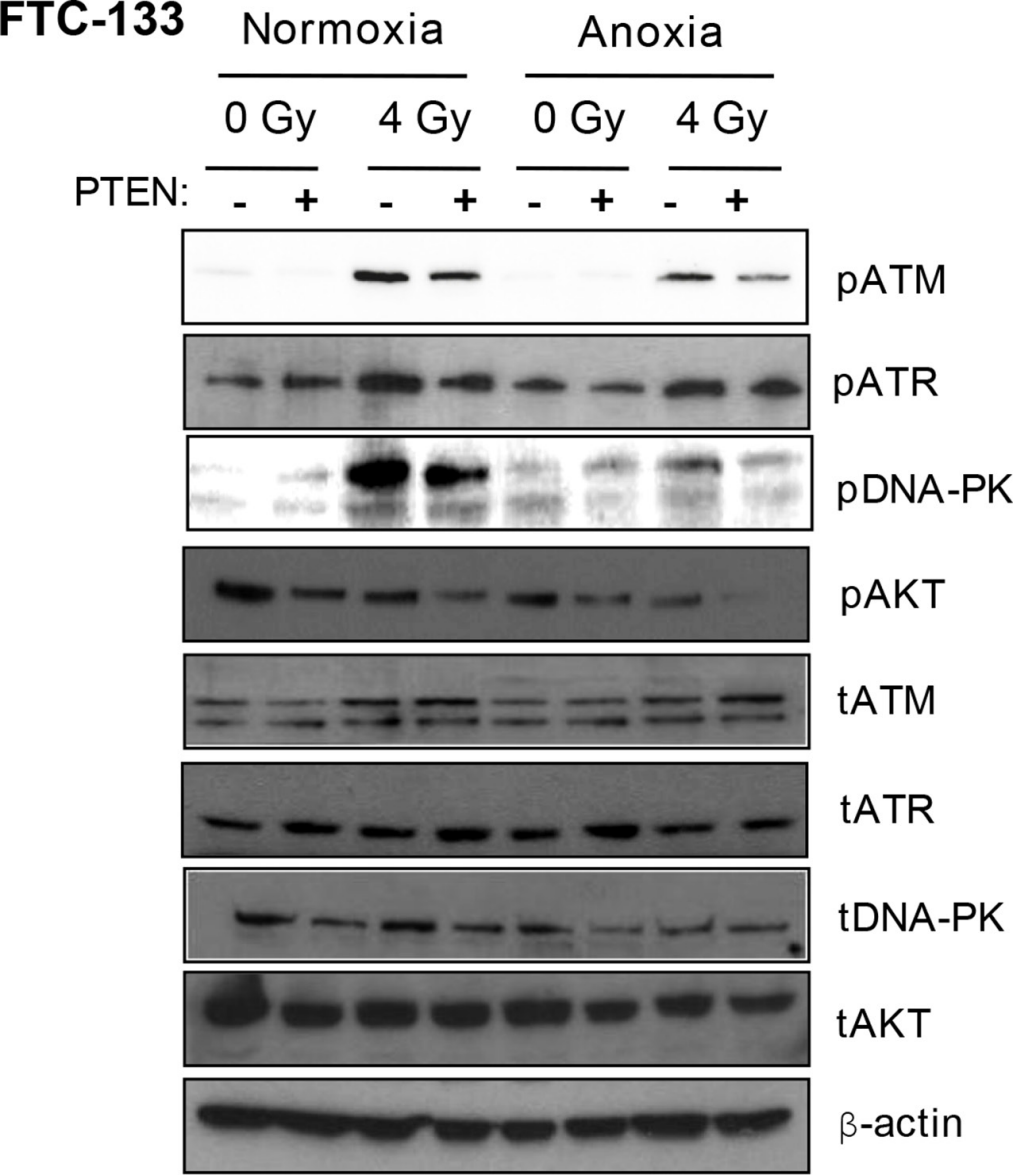
Under anoxia, genetic inhibition of PI3K inhibits radiation-induced ATM and DNA-PKcs activation PIKK activation was assessed via analysis of phospho(p)PIKK protein expression. Genetic inhibition of PI3K (via PTEN reconstitution into PTEN-null FTC-133 cells) had little effect on pATR expression under normoxia and anoxia. Radiation-induced pATM and pDNA-PKcs were inhibited in anoxic PTEN-expressing cells. PCI NEO and PTEN reconstituted FTC-133 cells were incubated in normoxia/anoxia for 18 h irradiated (4 Gy) whilst remaining in the specified conditions and lysates collected 1 h later for analysis. Cropped blots are representative of 4 independent experiments.

FTC-133 cells have hyperactive PI3K activity by virtue of PTEN loss, a commonly observed event in thyroid and other cancers. We used cell variants with reconstituted PTEN to enable comparison of pharmacological and genetic inhibition of the PI3K pathway: Western blotting of pPIKK in the paired cells did not phenocopy GDC-0941-mediated effects on pATR. However, as observed with GDC-0941 treatment, radiation-induced pATM and pDNA-PKcs activation was compromised in PTEN reconstituted cells, following irradiation of anoxic cells suggesting dependency on PI3K (Figure 2).

### Pharmacological and genetic inhibition of PI3K prolongs radiation-induced DNA damage and reduces clonogenic survival in anoxic FTC and ATC cells

**Figure 3:**
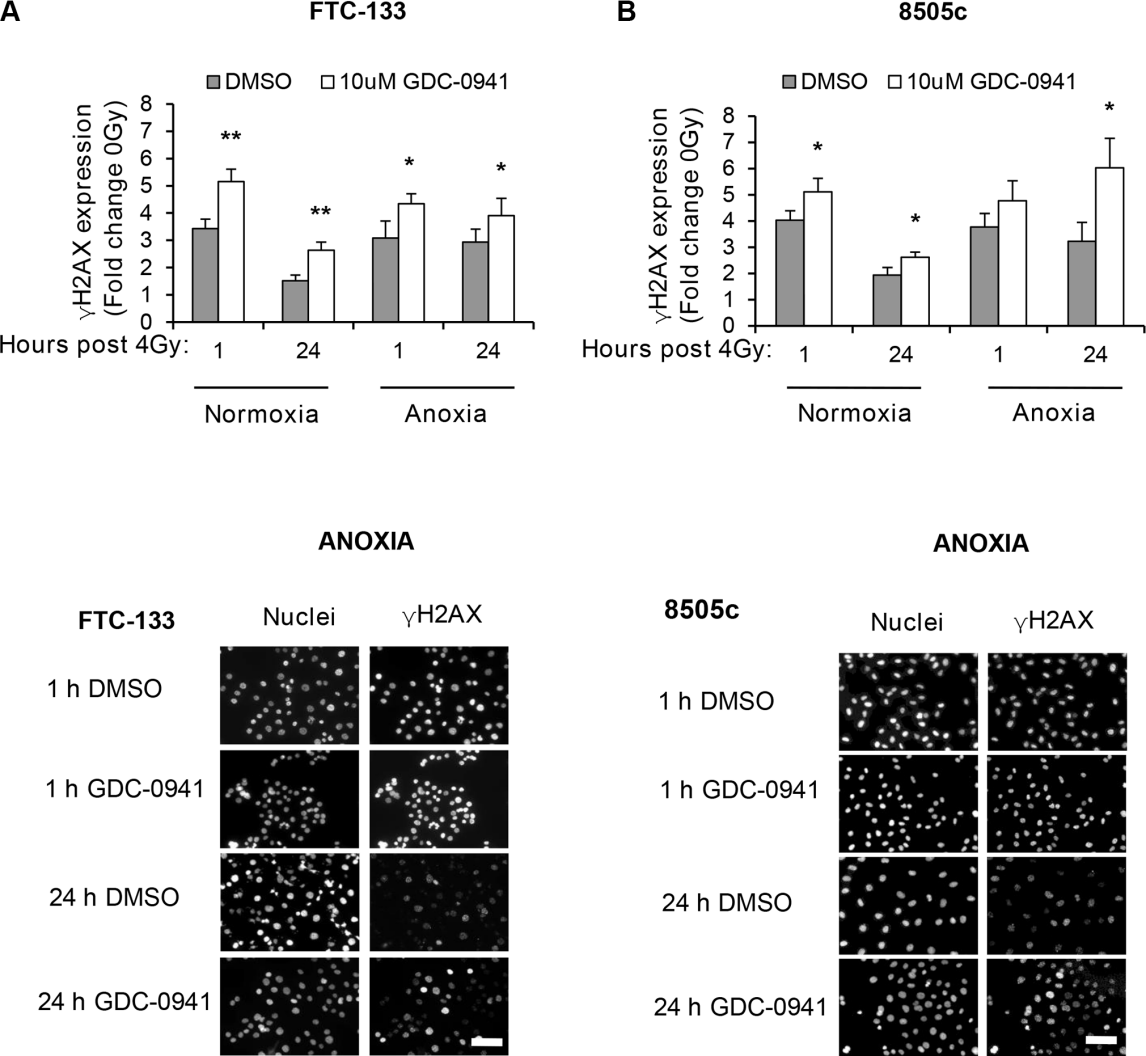
Pharmacological inhibition of PI3K increases and prolongs radiation-induced DNA damage across oxygen environments (**A**–**B**) GDC-0941 increases γH2AX expression 1 and 24 h post irradiation under normoxia and anoxia (*p < 0.05, **p < 0.01 compared to vehicle (DMSO) in same condition). Examples of immuno-fluorescence images of γH2AX under anoxia are shown. FTC-133 and 8505c cells were incubated under normoxia/anoxia for 18 h with DMSO or 10 μM GDC-0941 and irradiated (4 Gy) in the specified conditions. Samples remained in the specified conditions until fixation 1 and 24 h post 4 Gy. DNA damage was assessed by expression of γH2AX that was displayed as fold change of 0 Gy DMSO for each condition. Nuclei were counterstained with DAPI. Scale-bar 10 μm. Data represents the mean ± S.D. of 3 independent experiments.

**Figure 4:**
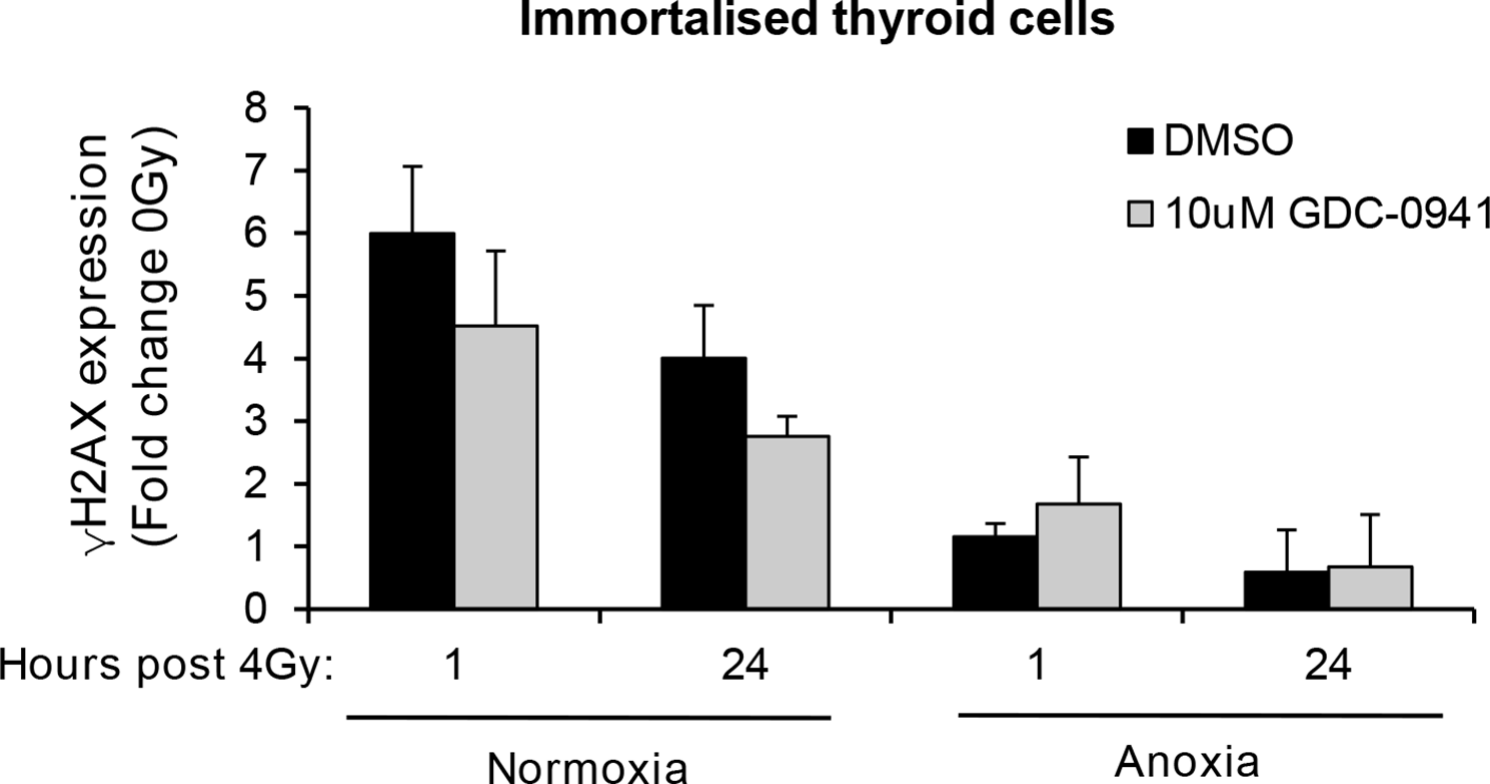
GDC-0941 does not affect radiation-induced DNA damage in immortalised thyroid cells Radiotherapy increased γH2AX expression to similar levels to that observed in thyroid carcinoma cell lines but had little effect under anoxia. GDC-0941 had little effect on radiation-induced DNA damage under normoxia or anoxia. Immortalised thyroid cells were treated and analysed as described in figure legend 3. Data represents the mean ± S.D. of 3 experimental repeats.

**Figure 5:**
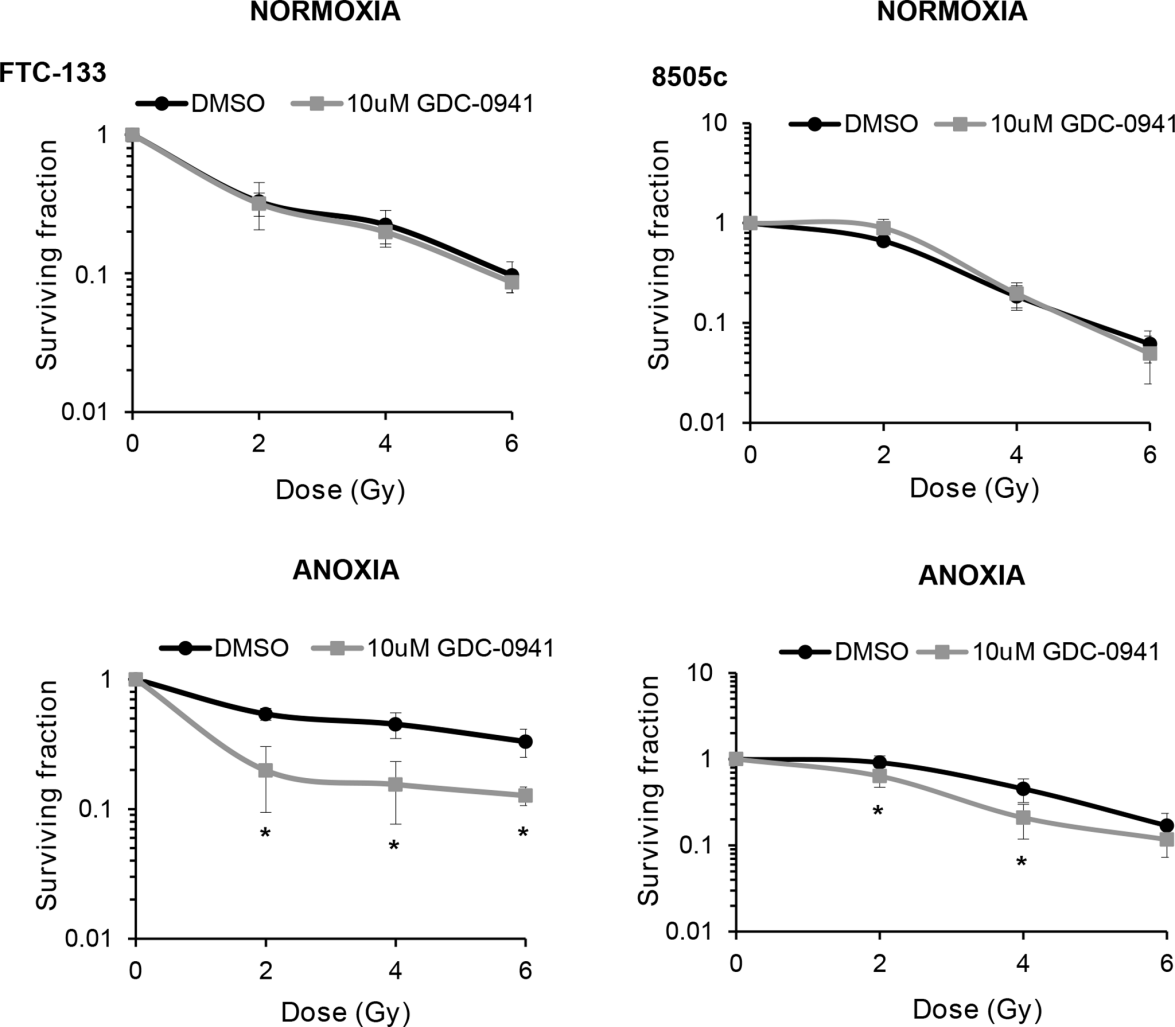
GDC-0941 reduces clonogenic survival of anoxic cells GDC-0941 had little effect on clonogenic survival under normoxia but significantly inhibited survival under anoxia (*p < 0.05 versus DMSO). For clonogenic assays, cells were incubated under normoxia/anoxia for 18 h with DMSO or 10 μM GDC-0941and irradiated (4 Gy) in the specified conditions. Samples remained in the specified conditions for 24 h post irradiation, then plated in serial dilution and cultured until visible colonies formed. Data represents the mean ± S.D. of 3 independent experiments.

**Figure 6:**
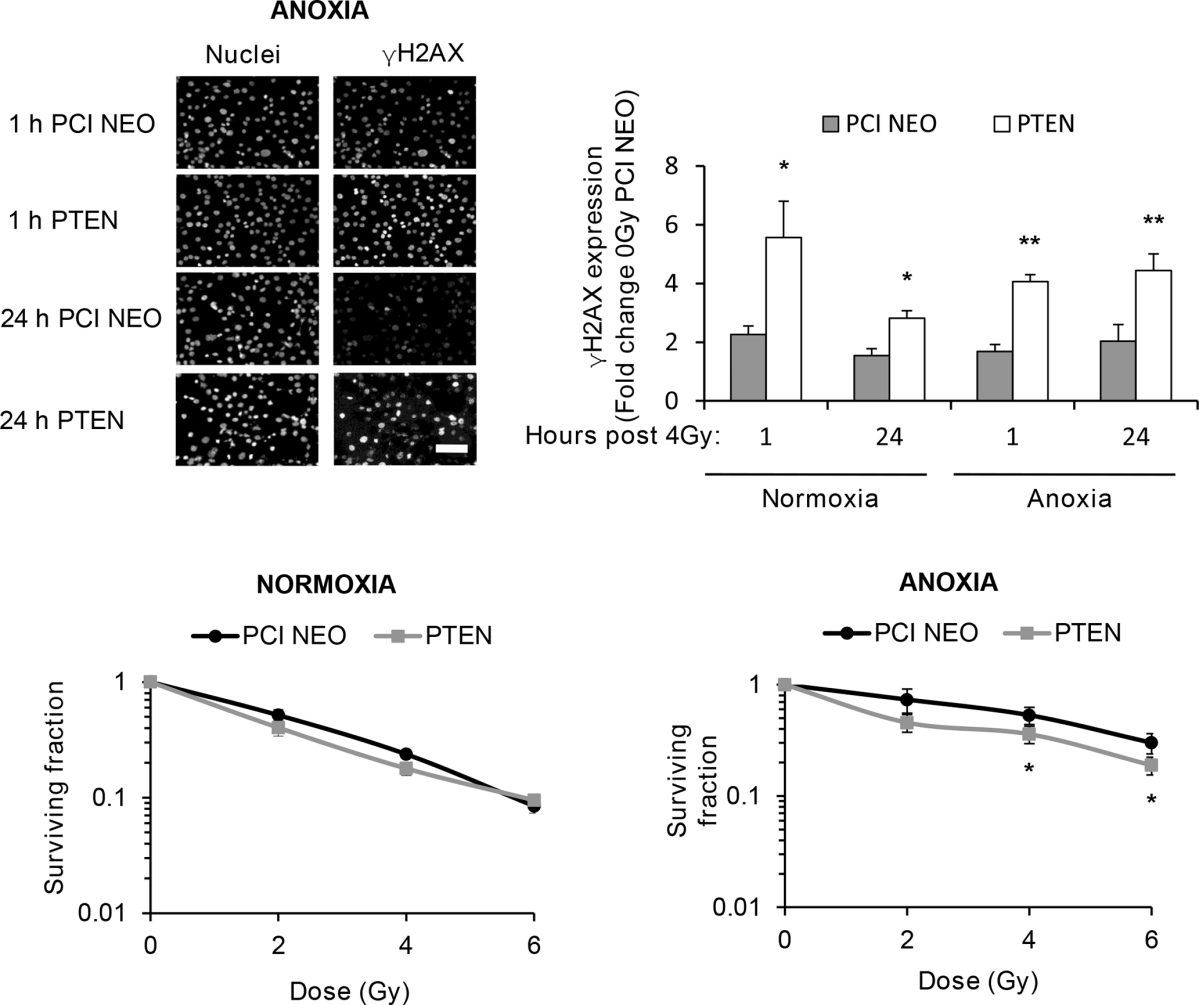
Genetic inhibition of PI3K increases and prolongs radiation-induced DNA damage across oxygen environments but selectively inhibits clonogenic survival of anoxic cells PTEN reconstitution in FTC-133 cells significantly increased γH2AX expression 1 and 24 h post irradiation under normoxia and anoxia (*p < 0.05, **p < 0.001) and reduced clonogenic survival under anoxia (*p < 0.05 versus DMSO), whilst having little effect under normoxia. PCI NEO and PTEN reconstituted FTC-133 cells were incubated under normoxia/anoxia for 18 h and irradiated (4 Gy) under condition. Cells then remained in normoxia/anoxia and were either fixed 1 and 24 h post 4 Gy for analysis of γH2AX expression or seeded in serial dilution 24 h post 4 Gy, and cultured until visible colonies formed for clonogenic survival assays. DNA damage was assessed by expression of γH2AX and displayed as fold change of 0 Gy PCI NEO for each condition. Nuclei were counterstained with DAPI. Scale-bar 10 μm. Data represents the mean ± S.D. of 3 independent experiments.

To determine if the observed effects of PI3K on PIKK activation influenced cellular response to radiotherapy, we evaluated DNA damage through induction and persistence of γH2AX expression following irradiation in normoxia and anoxia. In both conditions, GDC-0941 increased and prolonged γH2AX expression (Figure 3). Experiments were repeated using immortalised normal thyroid cells and here GDC-0941 had no effect on γH2AX expression over that observed with radiation alone (Figure 4). The persistence of DNA damage at prolonged times post-radiotherapy is commonly an indicator of the extent of potentially lethal DNA damage repair. To ascertain whether these changes correlated with survival, cells remained exposed to drug for 20 h following irradiation and were then seeded for clonogenic survival assays. Surprisingly, for both FTC-133 and 8505c cells irradiated under normoxia, the observed persistence of elevated γH2AX expression in GDC-0941 treated cells did not translate to reduced clonogenic survival (Figure 5). As anticipated, under anoxia, vehicle-treated cells displayed radioresistance compared with cells exposed to radiation in normoxia conditions. GDC-0941 treatment caused sensitization to anoxic-radiotherapy in both cell lines, with much more pronounced enhancement in FTC-133 cells (Figure 5). The effects of GDC-0941 on the extent and prolongation of γH2AX expression were phenocopied by PTEN reconstitution in FTC-133 cells. Again no significant effects of PI3K inhibition via PTEN were observed in terms of clonogenic survival following radiation treatment in normoxia (Figure 6). Significant sensitization was observed under anoxia, but interestingly, the magnitude was much reduced compared to GDC-0941 treated wildtype cells and mirrored more that observed in the 8505c model.

### GDC-0941 abrogates radiation-mediated effects on cell cycle

**Figure 7:**
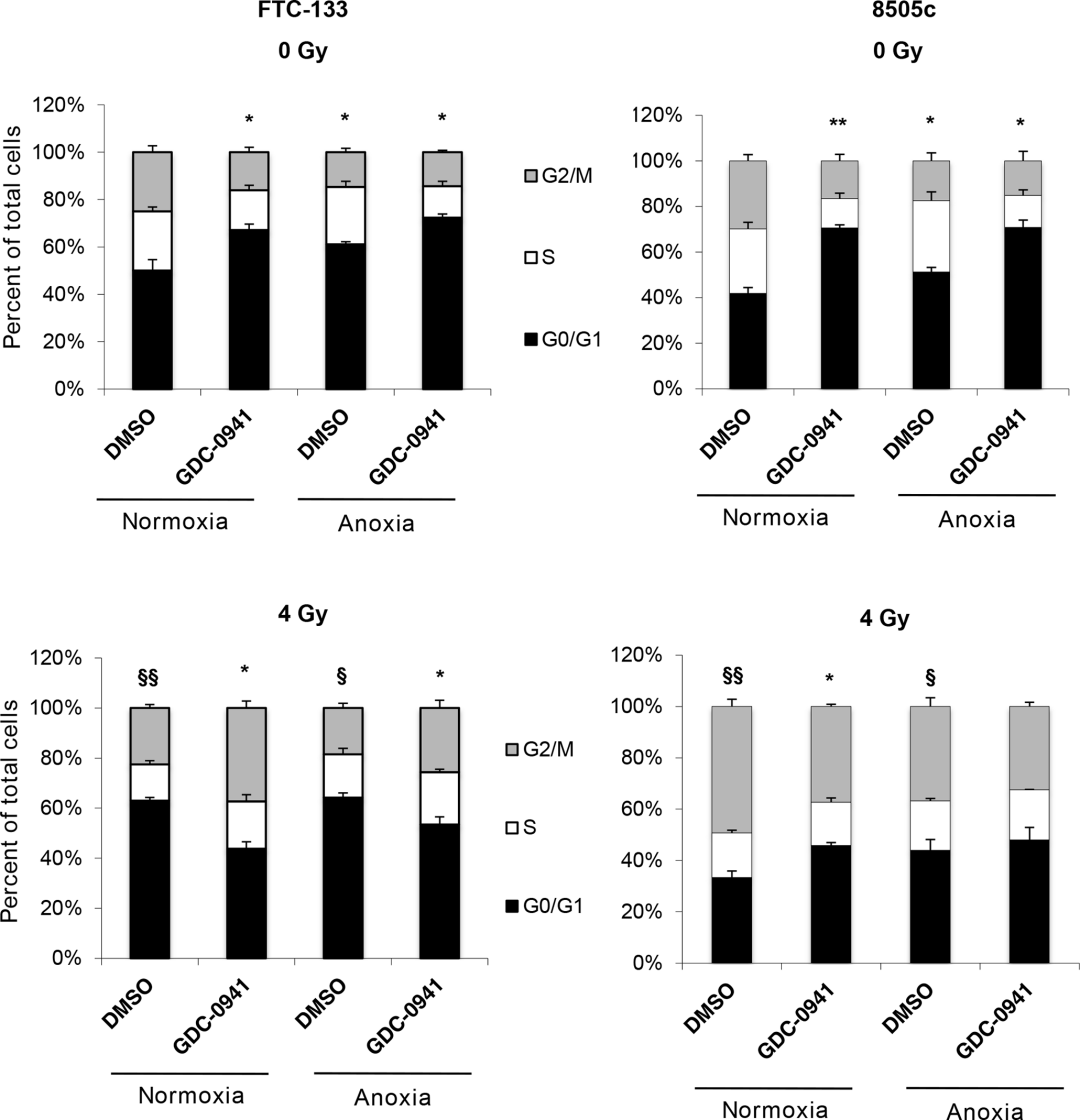
GDC-0941 abrogates radiation-mediated effects on cell cycle In un-irradiated cell lines, GDC-0941 and anoxia independently increased percentage cells in G0/G1 (*p < 0.05, **p < 0.01). Radiation had differential effects across cell lines; significantly increasing percentage cells in G0/G1 (FTC-133) and G2/M (8505c) under normoxia and anoxia (^§^*p* < 0.05, ^§§^*p* < 0.01). In irradiated FTC-133 cells, GDC-0941 significantly reduced percentage cells in G0/G1 under normoxia and anoxia (*p < 0.05). In irradiated 8505c cells, GDC-0941 significantly reduced percentage cells in G2/M under normoxia (*p < 0.05), with similar affects observed in anoxia. As for the clonogenic assays, FTC-133 and 8505c cells were incubated under normoxia/anoxia for 18 h with DMSO or 10 μM GDC-0941 and irradiated (4 Gy) in the specified conditions. Samples remained in the specified conditions for 24 h post irradiation, but instead of seeding for clonogenicity, samples were fixed and cell cycle distribution was assessed by flow cytometry analysis of propidium iodide stained cells. Data represents the mean ± S.E.M. of 3 independent experiments.

FTC-133 and 8505c cells were treated as for the clonogenic assay but instead of seeding for clonogenicity, cells were fixed and cell cycle distribution analysed by flow cytometry. GDC-0941 induced pronounced G1 phase arrest in both cell lines in both normoxic and anoxic conditions (Figure 7). The effect was markedly greater than the increased proportion of cells observed in the G1 phase following exposure to anoxia alone. Radiation had differential effects in FTC-133 and 8505c cells, inducing G1 and G2 arrest respectively after irradiation in normoxic and anoxic conditions (Figure 7). Combination of GDC-0941 clearly abrogated the cell cycle arrest observed in FTC-133 cells in both normoxia and anoxia. Similarly, the pronounced cell cycle changes observed in 8505c cells treated with either radiation or GDC-0941 alone were abrogated in co-treated cells. As detailed above, in both models, GDC-0941 inhibits activation of ATR following radiation treatment irrespective of oxygen condition. In previous studies direct targeting of ATR results in abrogation of cell cycle checkpoints following radiation treatment [18], consistent with the changes observed here, which may be associated with the marked inhibition of ATR activity.

### Effects of GDC-0941 on PIKK protein activation is maintained in irradiated human FTC-133 xenografts

**Figure 8:**
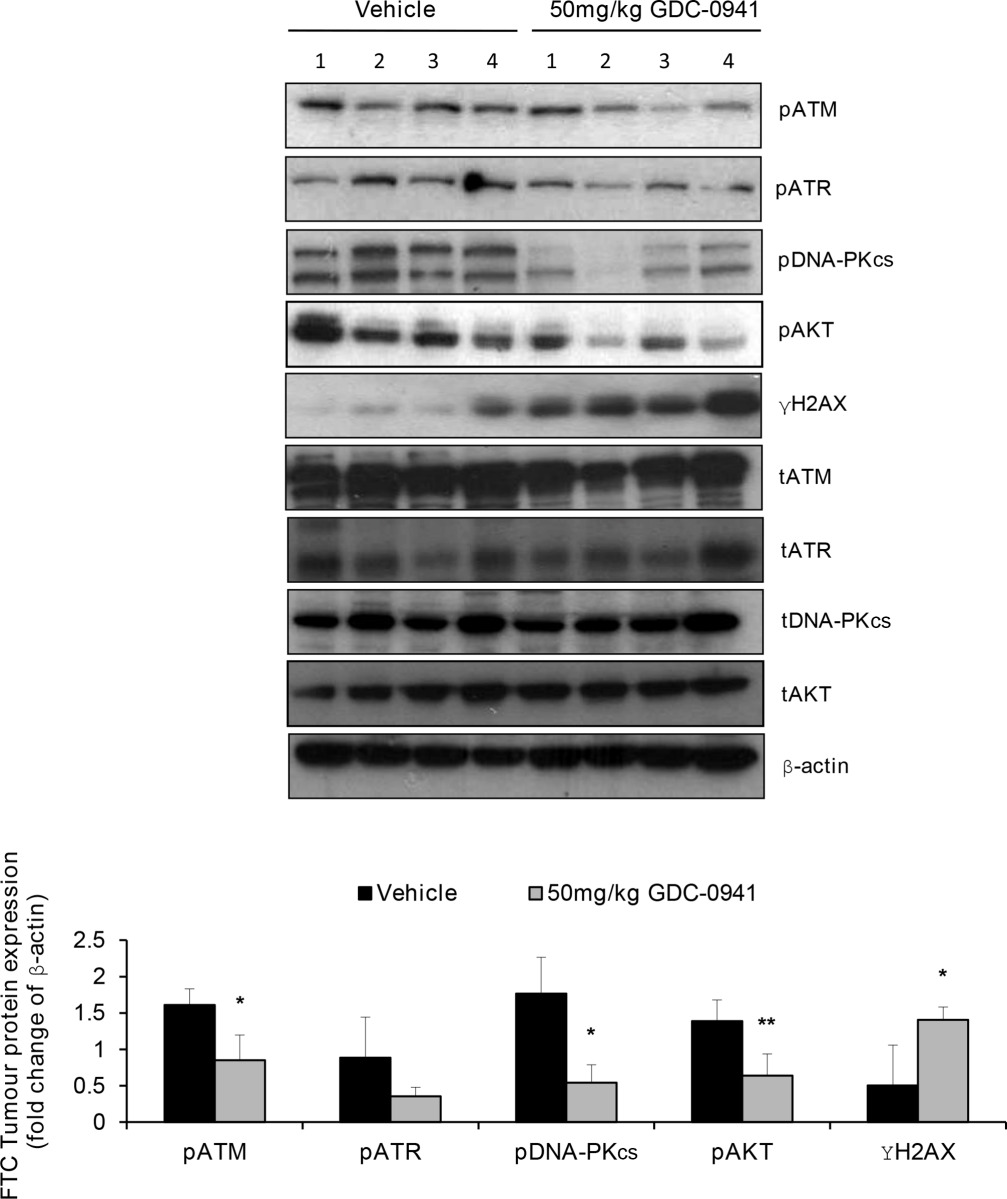
GDC-0941 inhibits PIKK activation and increases DNA damage in irradiated human FTC xenografts In irradiated FTC133 xenografts, GDC-0941 reduces pATR expression and significantly reduces pATM and pDNA-PKcs expression (*p < 0.05), whilst γH2AX expression is significantly increased (*p < 0.05). Inhibition of PI3K was confirmed by analysis of pAKT expression, which was reduced in GDC-0941 versus vehicle treated tumours (**p < 0.01). Mice received vehicle or 50 mg/kg GDC-0941 (p.o.) 4 h prior and immediately after radiotherapy (10 Gy fractionated (2 Gy) over 5 consecutive days). 1 h after the final dose, tumours were rapidly excised and immediately snap frozen for analysis of PIKK activity by western-blot of pPIKK expression. Cropped blot represents irradiated tumours from 4 independent mice that received either vehicle or GDC-0941. Graph represents densitometry data for the calculated mean ± S.D of the depicted western-blot.

We have previously reported that GDC-0941 treatment significantly reduces the growth of FTC-133 tumours and has pronounced anti-metastatic effects [14, 19]. To determine if the GDC-0941 mediated effects on PIKK activation translated *in vivo*, we first ascertained total PIKK expression by IHC in mice bearing human FTC xenografts. Interestingly, we found that out of 16 FTC xenografts analysed 4.5% and 18.2% of FTCs displayed complete loss of ATM and DNA-PKcs respectively, (data not depicted). FTC-133 tumours were treated with 2 Gy fractions of radiation over 5 consecutive days and dosed with 50 mg/kg GDC-0941 or vehicle twice daily, 4 h prior and immediately post radiation. Tumours were excised and snap frozen 1 h after the final dose and assessed for pPIKK expression. GDC-0941 significantly inhibited PI3K activity ascertained by pAKT expression in irradiated xenografts. Similar to *in vitro* observations GDC-0941 significantly inhibited pATM and pDNA-PKcs expression, and reduced expression of pATR, which coincided with a significant increase in γH2AX expression compared with tumours treated with radiation alone (Figure 8).

### GDC-0941 significantly improves the outcome of radiotherapy in human FTC-133 xenografts

**Figure 9:**
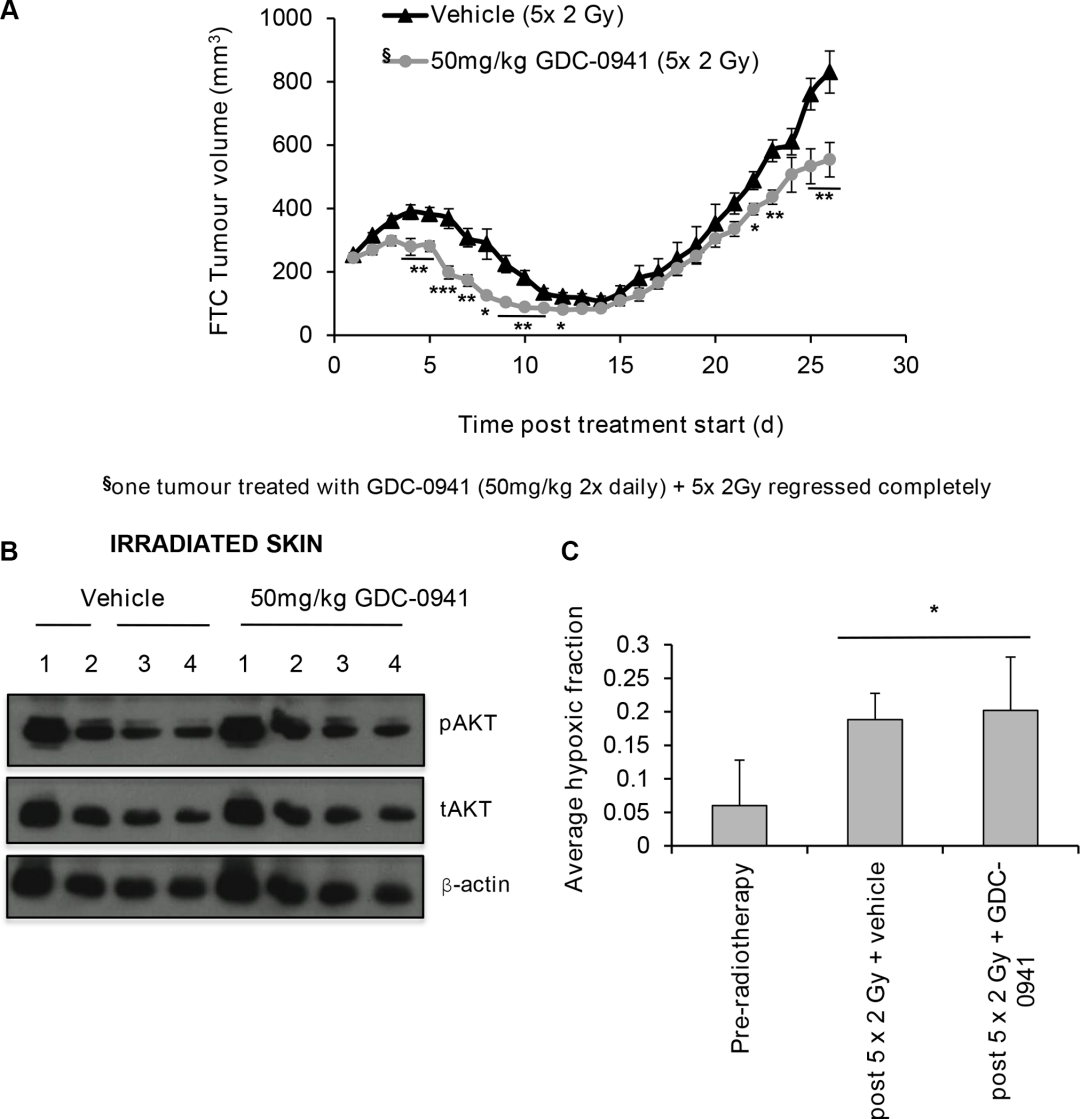
GDC-0941 increases tumour regression post-radiotherapy Mice were dosed twice daily (p.o.) with vehicle or 50 mg/kg GDC-0941 for 14 d. Tumours received 10 Gy radiation fractionated into 2 Gy over 5 days (treatment days 1-5). On these days vehicle/GDC-0941 was administered 4 h prior and immediately after radiation. (**A**) Mice dosed with GDC-0941 had significantly smaller tumour volumes on days 4–12, 22,23,25 and 26 (*p < 0.05, **p < 0.01, ***p < 0.001 versus vehicle treated mice). Data displayed shows up until the time when vehicle treated mice reached the designated end-point of three times the relative tumour volume (RTV3: 26.5 ± 5 days for radiation alone versus 31.5 ± 5 days for GDC-0941/radiation; *p* < 0.05). Data shown is for 11 vehicle and 9 GDC-0941 treated mice, Data represents the mean ± S.D. ^§^One co-treated tumour regressed completely and was excluded from analysis. (**B**) Mice received vehicle or 50 mg/kg GDC-0941 (p.o.) 4 h prior and immediately after radiotherapy (10 Gy fractionated (2 Gy) over 5 consecutive days). 1 h after the final dose, skin exposed to radiation (that covering the tumour) was excised, snap frozen and homogenised in lysis buffer for western-blot analysis. GDC-0941 did not affect pAKT expression in skin tissue exposed to radiotherapy. Cropped blot shows pAKT, tAKT and the loading control β-actin for mice that received radiation with either vehicle (*n* = 4) or with GDC-0941 (*n* = 4). (**C**) Tumour hypoxia was measured by IHC analysis of pimonidazole, dosed at 60 mg/kg (i.p.) 2 h prior to tumour excision. A significant increase in hypoxic fraction was observed in irradiated vehicle and GDC-0941 treated tumours (*p < 0.05 versus un-irradiated). GDC-0941 had no significant effect on hypoxic-fraction compared to vehicle in irradiated xenografts. Tumour volume did not significantly differ between groups. Data represents the mean ± S.D. of 4-5 mice per group.

To explore the effect of combining GDC-0941 with radiotherapy on tumour growth, mice bearing FTC-133 xenografts were treated with 2 Gy fractions over 5 consecutive days combined with 50 mg/kg GDC-0941 or vehicle (twice daily) for 14 days. GDC-0941 significantly improved radiotherapy outcome compared to vehicle treated controls, with one tumour showing complete regression (Figure 9A). GDC-0941 increased post-radiation tumour regression, and significantly increased the time taken for tumours to triple in size (RTV3: 26.5 ± 5 days for radiation alone versus 31.5 ± 5 days for the dual treatment; *p* < 0.05). Importantly, GDC-0941 did not affect pAKT expression in irradiated skin that was covering the tumour (Figure 9B), suggesting that at the dose given, GDC-0941 selectively radiosensitises tumour cells, and does not radiosensitise normal tissue.

Elegant studies have revealed that an alternate mechanism by which PI3K targeting strategies can enhance tumour response to radiotherapy is by reducing oxygen consumption within cells, consequently leading to a reduced hypoxic fraction within treated tumours [20]. As the level of hypoxia in tumours associates with the extent of radiotherapy resistance, we analysed hypoxia in tumours at treatment size and after the 5^th^ 2 Gy dose of radiation, with and without co-incidental GDC-0941. To enable this assessment, mice were treated with pimonidazole, a hypoxic marker, prior to tumour excision. Tumour hypoxia was assessed via IHC for pimonidazole adducts. Irradiated tumours had a significantly higher hypoxic fraction versus unirradiated controls. No significant differences in hypoxic fraction were observed in GDC-0941 versus vehicle treated irradiated tumours (Figure 9C). This was not due to tumour size as this did significantly differ between the two groups at the excision time point (456 ± 132 mm^3^ (vehicle-treated) versus 342 ± 82 mm^3^ (GDC-0941-treated)).

### PIKK proteins are a viable target in human thyroid cancers

**Figure 10:**
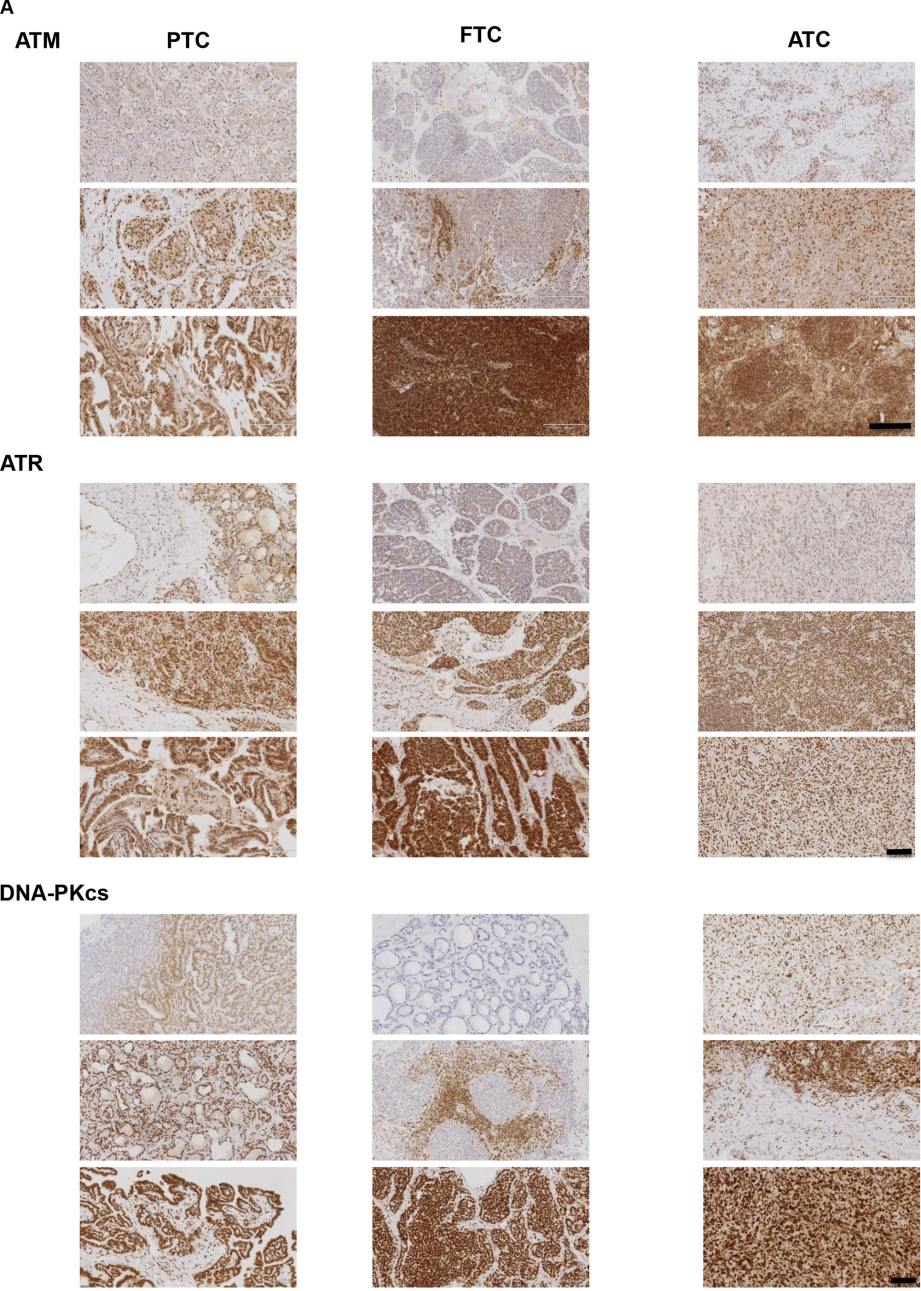

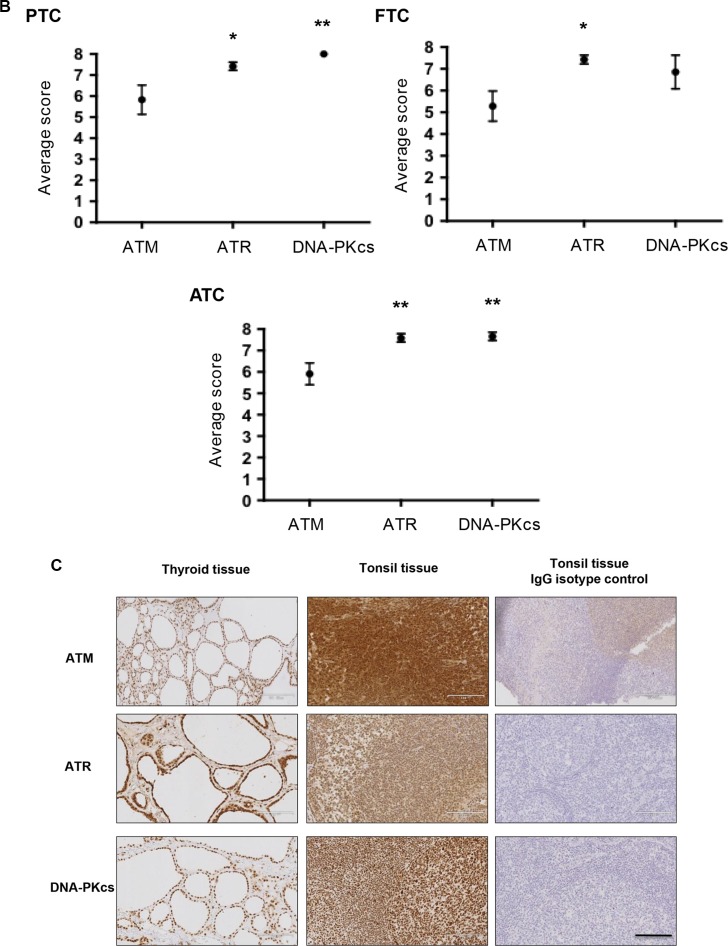
The PIKKS are a viable target in human thyroid cancer (**A**) Representative images of PIKK staining (brown). Intensity and distribution of PIKK proteins varied within each tumour classification. Representative images from three independent tumours are shown. Scale bar 1700 μm (for ATR and DNA-PKcs images) and 2000 μm (for ATM images). (**B**) PIKK expression was scored based on intensity and proportion of staining (described in methods). ATR and DNA-PKcs expression was significantly higher versus ATM in PTCs/ATCs and ATR expression significantly higher versus ATM in FTCs (*p < 0.05, **p < 0.01). Data represents mean ± S.E.M. of 12 PTC/ATC and 13 FTC. (**C**) Thyroid tissue stained positive for ATM, ATR and DNA-PKcs. Human tonsil was used as a known positive control for PIKK expression. Additionally tonsil tissue was exposed to the appropriate concentration matched IgG isotype antibodies as a negative control. See methods for full details. Scale bar 119 μm.

To ascertain the expression of PIKK proteins within and between classifications of human thyroid tumours, IHC was performed on 12 tumours representative of PTC and ATC and 13 FTCs. Intensity and distribution of the PIKK proteins differed from very weak to very intense nuclear staining. Distribution also varied from very diffuse to more focal isolated regions of staining (Figure 10A). Interestingly other cell types within the tumours varied for PIKK staining: stromal cells appeared to be negative for PIKK proteins, whereas tumour lymphocytes expressed all PIKK proteins but staining varied from weak to intense. Skeletal muscle that had been infiltrated by tumour cells was mainly negative for ATM/ATR but some staining was seen for DNA-PKcs (data not shown). Both ATR and DNA-PKcs expression was higher than ATM in all tumour classifications (Figure 10B). All ATCs examined expressed all of the PIKK proteins. ATM was expressed in 91.7% and 85.7% of PTCs and FTCs, and DNA-PKcs in 85.7% of FTCs analysed. Normal thyroid tissue was positive for the PIKK panel, with expression limited to the epithelium cells of the follicles lining the colloid space. Human tonsil was used as a known positive control for the PIKKs and concentration-matched isotype IgG antibodies were used on tonsil tissue for negative controls (Figure 10C).

## DISCUSSION

Currently, no conventional combined chemo/radiotherapies exist that significantly prolong life in patients with aggressive, radioresistant FTCs and ATCs [3]. Furthermore, the underlying molecular mechanisms contributing to these phenotypes are complex and remain poorly understood. A key pathway that contributes to thyroid cancer progression and radioresistance is the PI3K pathway [1, 7]. Here we explored the inter-relationship between PI3K and the PIKK proteins in irradiated thyroid cancer cells and determined if this differed depending on the oxygen environment. This is important since most solid tumours including thyroid contain regions of hypoxia; a condition fundamentally associated with radioresistance.

The PIKK proteins share significant sequence homology with PI3K [21, 22] therefore drugs targeting PI3K may also inhibit PIKK activity. Here we propose that inhibition of radiation-induced ATR is a GDC-0941 associated effect and not affected by oxygen tension. This is because whilst ATR activation was markedly inhibited by GDC-0941 in both cell lines, no effect on ATR activation was seen on PTEN reconstitution in FTC cells.

In contrast, we propose that radiation-induced ATM and DNA-PKcs activation has dependency on PI3K under anoxia; shown by inhibition of pATM and DNA-PKcs in both GDC-0941 treated and PTEN reconstituted cells, when compared to effects in normoxia. Further, this effect was more pronounced in FTC cells compared to that observed in 8505c cells: FTC133 cells harbour loss of PTEN, whereas 8505c cells predominantly carry a BRAF^V600^ mutation with some co-reliance on PI3K (described in [19, 23]). As effects on radiation-induced ATM and DNA-PKcs activation were more marked in the FTC line, this links a thyroid model that is predominantly driven by PI3K to a regulatory effect on ATM and DNA-PKcs activation.

It is known that at concentrations used in the functional studies (10 μM) GDC-0941 directly inhibits DNA-PKcs [17], and in accord with this we observed a dose-dependent reduction in DNA-PKcs activity in both cell lines. However, we observed inhibition of DNA-PKcs activity on PTEN reconstitution suggesting that although GDC-0941 may directly inhibit DNA-PKcs in these studies, there is also a dependency on PI3K for radiation-induced activation.

From our observations, the combined inhibitory effect of anoxia and GDC-0941 on PIKK activation was much greater than GDC-0941 alone. This may provide a mechanism for why GDC-0941 only reduced clonogenic survival in anoxic cells. Whilst reduced clonogenic survival was also observed in anoxic PTEN-expressing FTC133 cells; the magnitude of radiosensitisation was much less than with GDC-0941. This may reflect differences in GDC-0941 associated effects (inhibits all PIKKs) versus PI3K associated effects on ATM and DNA-PKcs activity only.

GDC-0941 abrogated radiation-induced cell cycle arrest. It has been shown that direct inhibition of ATR leads to abrogation of cell cycle checkpoints via modulation of Chk1 [18, 24]. Therefore, the marked inhibitory effect by GDC-0941 on ATR may contribute to the abrogation of radiation-mediated cell cycle arrest in all conditions. Since PI3K-inhibition only reduced clonogenic survival under anoxia, we postulate that PI3K-inhibition combined with radiation may lead to a cytostatic outcome under normoxia, but a cytotoxic effect under anoxia. In anoxia, the G2 arrest is less pronounced, and activity of all PIKKs is inhibited; ATM also contributes significantly to cell cycle control [24, 25]. Therefore the combination of these effects may account for the radiosensitisation observed under anoxia.

Collectively, these data suggest that GDC-0941 works by ablating a number of key cellular responses to radiation damage: DNA repair (via inhibition of ATM, ATR and DNA-PKcs activity), cell cycle (via inhibition of ATR and ATM) and cell survival (via PI3K inhibition).

To determine if this translated into a more realistic setting, mice bearing human FTC xenografts were treated with radiotherapy/GDC-0941 and effects on pPIKK expression and tumour growth explored. Importantly the GDC-0941 mediated inhibitory effects on radiation-induced PIKK activation translated *in vivo* in FTC133 xenografts leading to a significant radiosensitisation: Tumour growth delay was increased post radiotherapy in GDC-0941-treated mice. Since hypoxia promotes radioresistance it is important to highlight that cotreatment with GDC-0941 firstly did not radiosensitise normal thyroid cells or exposed skin, which likely reflects differences in PI3K hyperactivity and secondly, did not enhance radiation-induced increases in hypoxic fraction. Clinically these data highlight two important therapeutic characteristics; that GDC-0941 does not radio-sensitise non-cancerous tissue but selectively targets cancerous cells, and does not increase the radioresistant hypoxic fraction.

We show that the PIKK proteins are viable therapeutic targets in human thyroid cancers, as PIKKs were highly expressed across classifications, with ATM scoring consistently lower. Expression did not significantly differ between classifications but interestingly, some loss of PIKK proteins was observed in PTCs and FTCs. Loss of certain PIKKs may additionally open up new therapeutic strategies by virtue of a synthetic lethality approach. For example, we found that ATM was lost in 8.3% of PTCs and 14.3% of FTCs. Since one of the major DNA repair mechanisms, homologous recombination, is compromised due to loss of ATM in these tumour subsets; this may drive reliance of these cancer cells on the remaining DDR pathways to survive inflicted DNA damage. Therefore inhibiting other key components of the DRR response such as ATR and DNA-PKcs may sensitise thyroid cancer cells to DNA damaging therapy. In accord with this, similar synthetic lethality approaches have been successful in a range of tumour types [26].

Interestingly other cell types within the tumours varied for PIKK staining. Thus, there was significant heterogeneity of PIKK expression within the different tumour–associated cell populations. These findings suggest that activation of PIKK proteins in other cells within the tumour microenvironment may also contribute to the DNA damage response and tumour cell survival post radiotherapy. Unfortunately, we were unable to detect pPIKK expression owing to technical problems with phospho-antibody staining.

In summary, inhibition of radiation-induced ATR activity is a GDC-0941 associated effect, whereas radiation-induced ATM and DNA-PKcs activation has dependency on PI3K, is associated more with hypoxia and is more pronounced in FTCs with a PTEN mutation: This links a thyroid model that is predominantly driven by PI3K, to a regulatory role on ATM and DNA-PKcs. From our observations both the ATM and DNA-PKcs only effect (reduced activation in irradiated PTEN mutant cells under anoxia) or the ATR only effect (reduced activation in irradiated 8505c cells treated with GDC-0941 under anoxia) causes some hypoxic radiosensitisation. However this is more pronounced if both the PI3K pathway and PIKK activation is abrogated, as seen with GDC-0941 in irradiated FTC cells, under anoxia.

Importantly, we show that these interactions between the PI3K pathway and PIKK activity exist in human xenografts and contribute to radioresistance. We further show that the PIKKs are viable therapeutic targets in human tumour samples. These data provide new insight into the mechanisms of radioresistance in thyroid carcinoma that has important therapeutic implications.

## MATERIALS AND METHODS

### Cell culture

FTC (FTC-133), ATC (8505c), and derivative cell lines are described previously [19]. FTC-133 cells stably expressing PTEN and PCI-NEO [27], kindly provided by Professor Oliver Gimm, University of Linkoping, Sweden) were cultured in media containing 1 mg/ml G418. Immortalized thyroid cells [28] were cultured in standard RMPI medium supplemented with 10% fetal calf serum and 2 mM L-glutamine. Low passage 8505c cells were used in all studies, purchased from DSMZ, Braunschweig, Germany. FTC-133 parental and variant cell lines were authenticated post studies by short tandem repeat (STR) profiling. The percentage match given against the STR profile from the European Collection of Authenticated Cell Cultures (ECACC) was 91.3%.

### Exposure to anoxia

Cells were gassed with 5% CO_2_:5% H_2_:90% N_2_ through a palladium catalyst in an anaerobic chamber (Sheldon Manufacturing, Cornelius, OR).

### Radiation treatments

Cells were irradiated (Faxitron X-ray; Faxitron Bioptics, Lincolnshire, USA) in sealed airtight containers under the appropriate O_2_-tension. X-ray doses were given at a rate of 0.952 Gy/min. Cells were then transferred back to the appropriate experimental condition for a specified time period depending on the study.

### Immuno-fluorescence and γH2AX quantification

Cells were fixed and permeablised 1 and 24 h post irradiation. GDC-0941 treated cells were maintained in drug for the times indicated post irradiation. Fixed cells were incubated with a primary antibody against γH2AX for 1h at 37°C, washed and incubated with a secondary antibody (AlexaFluor598: Life technologies Ltd, Paisley, UK) for 45 min at 37°C. Nuclei were counter-stained with 4′,6-diamidino-2-phenylindole (DAPI). γH2AX expression was visualised at 200X magnification on a Snapshot Widefield (Olympus BX51) upright fluorescence microscope using a 10×/0.30 Plan Fln objective and captured using a Coolsnap ES camera (Photometrics) and MetaVue Software (Molecular Devices). Pictures of three fields of view were taken totalling 100 cells per experiment. Average γH2AX expression was quantified by calculating average fluorescence intensity of γH2AX per nucleus. Expression was displayed as fold change of the untreated 0 Gy control in the relevant condition unless stated otherwise.

### Western-blotting

Protocols were used as previously described [29]. For PIKK analysis 70 μg protein was loaded per well on density gradient SDS-PAGE gels to allow detection of the lower molecular weight loading control protein β-actin on the same gel.

### Primary antibodies

Antibodies used were mouse monoclonals; γH2AX (Millipore (U.K.) Ltd, Watford, UK), Pimonidazole (Hydroxyprobe 1 clone 4.3.11.3, Natural Pharmacia International Inc, Burlington, USA), AKT-phospho (p) S473 (Cell Signaling Technology, CA, USA), and β-actin (Sigma-Aldrich, Gillingham, UK). Rabbit monoclonals used were: ATM-pS981, total (t)-ATM, DNA-PK-pS2056, tDNA-PK, Isotype control rabbit IgG (Epitomics, CA, USA). Rabbit polyclonals used were; ATR-pS428, tATR, tAKT (Cell Signaling Technology, CA, USA) and rabbit IgG (Vector Laboratories Ltd, Peterborough, UK).

### Clonogenic survival assays

Cell cultures were incubated in normoxia/anoxia for 4 h, irradiated and put back in condition (for stable clones) or immediately treated with 10 μM GDC-0941 or vehicle (DMSO)in condition, and left for a further 20 h. Cells were then plated at low seeding densities and cultured for 10 to 12 d. Colonies were stained with methylene blue and counted. Colony-forming efficiency was normalised to that of the appropriate control.

### Cell cycle analysis

Cells were treated as in the clonogenic assay. At the point of seeding for colony formation, cells were stained with propidium iodide solution, for cell cycle analysis as described [30].

### Xenograft studies

To initiate xenografts, FTC-133 cells were implanted sub-cutaneously (0.1 ml of a 5 × 10^7^/ml stock in PBS) in female nude mice (CBA *nu/nu*, aged 8–12 weeks, weight range; 20–27 g) and tumour volume measured at least five times weekly and weight daily. Mice were randomly assigned into treatment groups and radiotherapies commenced when FTC-133 tumours reached 200–250 mm^3^. GDC-0941 was prepared in 0.5% hydroxypropyl methylcellulose (vehicle) and administered by oral gavage (p.o.). GDC-0941 was dosed in 0.1 ml/10g body weight. Mice were dosed twice daily with vehicle or 50 mg/kg GDC-0941 combined with radiation (10Gy fractionated into 2 Gy over 5 consecutive days (d)). On radiation days, mice received the first dose 4 h prior to radiation and the second dose immediately after. For analysis of GDC-0941-mediated effects on the primary tumour, tumours were rapidly excised 1 h after the final dose of vehicle/drug and immediately snap frozen. For analysis of radiosensitization, dosing regimes continued for 9 d post radiotherapy. Tumours were excised at maximum burden (900–1000 mm^3^).

All procedures were carried out in accordance with the Animals in Scientific Procedures Act 1986 and in line with the guidelines for the welfare and use of animals in cancer 2010 [31]. Protocols were ethically approved by the University of Manchester Animal Welfare and Ethical Review Body and were conducted with the highest standard of welfare under the authority of Home Office project license 40/3112 (held by Professor K.J.W.).

### Patient samples

For the use of thyroid carcinoma tissue, patient informed consent and ethical approval was obtained from the Faculty of Medicine ethics commission board at the Martin Luther University of Halle-Wittenberg, Germany, as previously described [29]. The human tonsil tissue was sourced as an anonymous control from the Histology archive at the Cancer Research UK Manchester Institute, Manchester, UK.

### Immunohistochemistry (IHC)

12 PTC, 13 FTC and 12 ATC samples were histologically evaluated and used for analysis of total PIKK expression. Samples were dewaxed, rehydrated and antigen retrieval performed in a pascal pressure retriever (DAKO UK Ltd, Ely, UK) that heated samples to 125°C for 1 min, then 90°C for 10 min. Target retrieval solution pH9 and pH6 was used for ATM/DNA-PK and ATR respectively. Samples were incubated in hydrogen peroxide block solution (10 min at room temperature (RT)), washed and blocked with protein blocking solution (DAKO: 5 min at RT). ATM, ATR, DNA-PK and pimonidazole (Hydroxyprobe 1 clone 4.3.11.3) antibodies were diluted 1:200, 1:50, 1:25 and 1:50 respectively. Appropriate isotype control IgG antibodies were concentration matched to the primary antibody and used as negative controls. Human tonsil was used as a positive–staining control. Samples were incubated for 1 h at RT with primary antibody, washed and incubated with Envision system HRP labelled rabbit or mouse polymer (DAKO: 30 min at RT) and stained with diaminobenzidene solution (DAKO:10 min). Slides were counterstained with Mayer's haemotoxylin, dehydrated and mounted. Images were captured using a Leica SCN400 imaging analyzer (Leica Microsystems (UK) Ltd, Milton Keynes, UK). Samples were scored according to protocols adapted from [32] as described previously [29]. PIKK protein staining and expression was validated and scored by a consultant histopathologist. This scoring allowed for necrosis, tumour heterogeneity and lymphoid infiltration.

### Statistical analysis

One-way ANOVA with Tukey *post hoc* test and Student's *t* tests were used.
